# Recalcitrant urticaria controlled with a combination of mycophenolate mofetil and sulfasalazine

**DOI:** 10.1016/j.ijwd.2021.10.013

**Published:** 2021-11-11

**Authors:** Alyssa G. Ashbaugh, Jenny E. Murase

**Affiliations:** a Department of Dermatology, University of California, San Francisco, San Francisco, California; b Department of Dermatology, Palo Alto Foundation Medical Group, Mountain View, California

**Keywords:** Chronic urticaria, mycophenolate mofetil

 

**What is known about this subject in regard to women and their families?**

• Chronic urticaria is twice as common in women as in men.
• The chronic nature and discomfort associated with chronic urticaria is distressing and often exerts a negative effect on patients’ quality of life.
• Patients typically are treated in a stepwise fashion, layering many different medications on top of one another to find relief.
• Given that 50% to 70% of cases of chronic urticaria are idiopathic, therapies typically target symptoms rather than the underlying cause.

**What is new from this article as messages for women and their families?**

• Therapy targeting cell-mediated immunity, such as mycophenolate mofetil and sulfasalazine, may be helpful for patients who are recalcitrant to high-dose antihistamine medications and omalizumab.
• The combination of mycophenolate mofetil and sulfasalazine may be helpful in controlling chronic urticaria in cases recalcitrant to sulfasalazine alone.

*Dear Editors,*

A 54-year-old female patient with a history of asthma and allergic rhinitis presented with severe, recalcitrant, chronic urticaria for 12 years, for which she took up to 29 pills of antihistamines (varied combinations of diphenhydramine, hydroxyzine, cetirizine, loratadine, levocetirizine, famotidine, and ranitidine) daily with no side effects. She had previously failed treatment with montelukast, her hives worsened with omalizumab, and although hydroxychloroquine provided partial relief, the patient discontinued therapy due to unfavorable side effects. Even with high doses of antihistamines, she experienced diffuse edema and pruritus, as well as warmth and pain of her lips if she did not take a midnight dose of antihistamines. The patient denied any temporary improvement with intramuscular triamcinolone and short courses of prednisone.

Workup for mastocytosis and leukemia was negative. The patient had a Chronic Urticaria Index score of >50, a normal number of mast cells, and negative tryptase. Given that her number of mast cells was normal, yet she had an exceptionally positive Chronic Urticaria Index score, we suspected that her mast cells were overstimulated and conjectured that therapy targeting the cells stimulating the mast cells rather than the mast cells themselves may prove more efficacious in our patient. Thus, we started the patient on sulfasalazine 1000 mg daily and titrated up to as high as 2000 mg daily; however, she could not tolerate such higher doses due to diarrhea and nausea.

Given that the patient had experienced a partial response after 5 months on sulfasalazine 1000 mg daily, we continued her treatment with sulfasalazine 1000 mg daily and added mycophenolate mofetil (MM) 500 mg twice per day (BID). We then titrated MM up to 1000 mg BID 3 months later and up to 1500 mg BID 3 months after that. The patient experienced dramatic improvement within 3 weeks of initiating MM. When we held both medications 2 weeks before and after her COVID-19 vaccination, she experienced another unbearable flare. Upon restarting MM and sulfasalazine, the patient was extremely satisfied with her results and reported only taking two antihistamine pills per day for seasonal allergy purposes compared with the 29 antihistamine pills prior to MM therapy. She has been in remission now for 16 months and is currently not interested in decreasing either the sulfasalazine or MM.

MM is an immunosuppressive agent that has been used to treat dermatologic conditions such as psoriasis, atopic dermatitis, and urticarial vasculitis, and has been assessed for the treatment of severe, recalcitrant, chronic urticaria (Shahar et al., 2006). Sulfasalazine inhibits nuclear factor kappa B activation, which in turn both inhibits T cell inflammatory cytokine production and plasma cell antibody synthesis (Wahl et al., 1998), and has also been used to treat recalcitrant chronic urticaria. However, given the limited number of studies and patients treated in these studies, as well as the inconsistency in response, the strength of the recommendation has been designated as very poor for both agents. No negative effects were noted in patients with recalcitrant chronic urticaria who were treated with MM; however, one patient reported worsening of symptoms, and other side effects (e.g., leukopenia and rhabdomyolysis) have been reported in patients with recalcitrant chronic urticaria who were treated with sulfasalazine (Holm et al., 2018).

MM acts as an antimetabolite, preferentially depleting guanosine nucleotides in T and B cells and subsequently inhibiting their proliferation, cell-mediated immunity, and antibody production (Allison, 2005). Given that MM prevents lymphocyte recruitment, it may dampen the production of immunoglobulin-E antibodies, thus preventing mast cell overactivation in patients with chronic urticaria who do not respond to antihistamines, which only modulate downstream effects of mast cell activation. Sulfasalazine inhibits nuclear factor kappa B activation, which in turn both inhibits T cell inflammatory cytokine production and plasma cell antibody synthesis (Wahl et al., 1998).

This case of chronic urticaria recalcitrant to traditional and add-on therapies that ultimately responded to a therapy targeting cell-mediated immunity suggests that therapies that target pathways upstream of mast cell activity, such as MM, may prove efficacious in patients who are recalcitrant to the traditional step-wise approach treatment of chronic urticaria.
